# Translation and validation of the Sindhi version of the general medication adherence scale in patients with chronic diseases

**DOI:** 10.3389/fphar.2023.1235032

**Published:** 2023-09-20

**Authors:** Md. Ashraful Islam, Wajiha Iffat, Shahlla Imam, Sadia Shakeel, Abdul Rasheed, Atta Abbas Naqvi

**Affiliations:** ^1^ Department of Pharmacy Practice, College of Clinical Pharmacy, Imam Abdulrahman Bin Faisal University, Dammam, Saudi Arabia; ^2^ Department of Pharmaceutics, Dow College of Pharmacy, Dow University of Health Sciences, Karachi, Pakistan; ^3^ Department of Pharmacognosy Institute of Pharmaceutical Sciences Jinnah Sindh Medical University Karachi, Karachi, Pakistan; ^4^ Department of Pharmacy Practice, Dow College of Pharmacy, Dow University of Health Sciences, Karachi, Pakistan; ^5^ Institute of Pharmaceutical Sciences, Jinnah Sindh Medical University Karachi, Karachi, Pakistan; ^6^ Reading School of Pharmacy, University of Reading, Whiteknights Campus, Reading, United Kingdom

**Keywords:** medication adherence, patient preferences, questionnaire, validation studies, Sindhi, Pakistan

## Abstract

**Background:** There is no medication adherence scale available in Sindhi language currently. Hence, the Sindhi speaking population will either use a translator or provide their medical history in another language for documentation of medical conditions. This poses a challenge in monitoring and evaluating adherence to medications within this linguistic community.

**Aim:** The aim of this study was to translate and validate the Sindhi version of the General Medication Adherence Scale (GMAS-S) in patients with chronic diseases.

**Methods:** This was a cross-sectional study of 4 months duration and was conducted in out-patient department of a university affiliated hospital in Karachi, Pakistan. All adults with chronic diseases, who were on long-term medications, and able to read and understand Sindhi language were invited. Convenience sampling was employed and a questionnaire consisting of demographic questions and the Sindhi version of GMAS was used. The translation of the scale was carried out. Confirmatory factor analysis (CFA) was conducted, and a structural equation model (SEM) was developed. Fit indices, namely, goodness of fit index (GFI), adjusted goodness of fit index (AGFI), Tucker Lewis index (TLI), comparative fit index (CFI), and root mean square error of approximation (RMSEA) were reported. Reliability was assessed using Cronbach’s alpha (α), intraclass correlation coefficient (ICC), corrected item-to-total correlation (ITC) and item deletion. Data were analysed through IBM SPSS version 23 and IBM AMOS version 25. The study obtained ethical clearance.

**Results:** A total of 150 responses were analysed. The reliability of the Sindhi version of GMAS was (α) = 0.696. The intraclass correlation coefficient (ICC) was reported at 0.696 (95% CI: 0.618–0.763). The values for the fit indices were as follows: χ^2^/df = 1.84, GFI = 0.918, TLI = 0.920, CFI = 0.942, AGFI = 0.864, and RMSEA = 0.075. All values except AGFI were in the acceptable ranges and indicated good fitness. Most participants (80.7%) appeared non-adherent to their medications.

**Conclusion:** The results of the study demonstrate that the Sindhi version of the GMAS is a valid and reliable scale to measure adherence in Sindhi speaking persons with chronic diseases.

## 1. Introduction

Adherence to long-term medications for chronic illness is a global issue. Adherence to medication can be explained as the extent to which a patients’ dietary and medicine taking behavior, and lifestyle is consistent to the recommended instructions from a healthcare provider (Yousuf et al., 2023; World Health Organization WHO, 2003). Non-adherence to medication not only has clinical implications but is one of the core reasons for additional health expenditure owing to complications and hospital admissions. It was reported in a systematic review that the healthcare expenditure attributed to the non-adherence due to all causes was roughly in range between USD 5000 to USD 52,000 worldwide (Cutler et al., 2018). Due to the lack of literature, such figures for Pakistani patients have never been reported. However, it is reported that most patients in Pakistan bear their healthcare expenditure out-of-pocket. According to the World Bank data, approximately 55% of total healthcare expenditure in Pakistan was out-of-pocket in 2020 (The World Bank, 2023).

As per the figures for 2017 census from Pakistan Bureau of Statistics (PBS), the country has a population above 200 million (Pakistan Bureau of Statistics PBS, 2017a). Several studies have reported mixed results in terms of adherence to medications among patients with chronic illnesses in Pakistan. For instance, a study reported that 37.7% of patients who visited a clinic setting in Islamabad were non-adherent to their antihypertensive medications (Mahmood et al., 2020). Similarly, in another study, 36% of in-patients who attended a healthcare setting in Karachi were observed to be non-adherent (Yousuf et al., 2023). In another study, it was observed that a large proportion of patients with rheumatoid arthritis were adherent to their therapy (Naqvi et al., 2020a). However, the figures for adherence in this population have been reported by studies that have either used the English or Urdu versions of adherence scales (Naqvi et al., 2020a; Mahmood et al., 2020; Yousuf et al., 2023). Therefore, it can be argued that adherence for the patients who speak a language other than the former two languages such as Sindhi speaking patients have largely remained undocumented or under-reported.

According to the Pakistan Bureau of Statistics 2017 census results, the total population of individuals who indicated Sindhi as their mother tongue has crossed 29 million in the province of Sindh (Pakistan Bureau of Statistics PBS, 2017b). There is no medication adherence scale available in Sindhi language at the moment. This means that this sizeable population will either use a translator or provide their medical history in another language such as Urdu or English for documentation of medical conditions. This poses a challenge in monitoring and evaluating adherence to medications within this linguistic community. Hence, there is a need to translate and validate a Sindhi version of the scale.

The General medication adherence scale was developed in Urdu language for Pakistani patients (Naqvi et al., 2018). It has since then translated and validated in several languages such as English (Naqvi et al., 2019a), Arabic (Naqvi et al., 2020b; Maryem et al., 2023), Chinese (Wang et al., 2021), Vietnamese (Nguyen et al., 2021), and Nepalese (Shrestha et al., 2021). Therefore, to increase the availability of this scale in local population, the scale must be translated into Sindhi language. This would allow clinicians to assess medication adherence of Sindhi speaking patients with chronic illnesses. This would set a good precedent for other studies to translate this scale into other local and regional languages of the country. Therefore, the objective of this study was to translate and validate the Sindhi version of the GMAS in this population.

## 2. Methods

A cross-sectional study was conducted in the out-patient department (OPD) of a tertiary care hospital affiliated with Dow University of Health Sciences, in Karachi, Pakistan. This study was conducted from 18 February 2022 to the end of June 2022.

### 2.1. Participants and eligibility criteria

All adult patients, who were able to read, and understand Sindhi, diagnosed with a chronic disease such as diabetes, hypertension, and so on, at least 3 months before this study, and were prescribed with medications for long-term use, were eligible to participate in the study. Patients who had any acute conditions, had a planned surgery, and/or were pregnant, were not included in the study. In addition, those patients who did not consent to participate were excluded from the study.

### 2.2. Sampling and sample size estimates

The method of convenience sampling was used to gather data from eligible patients. The sampling was based on the approach of a study that for the confirmatory factor analysis (CFA) model, a total of 150 samples with normal distribution and without any missing data, may provide a power of 0.81 (Muthén and Muthén, 2000). Therefore, this study involved collecting at least 150 surveys that had normally distributed data and no missing values. A total of 150 surveys without any missing data were collected.

### 2.3. Questionnaire

The questionnaire used in this study contained two sections. The first section was a demographic form that contained questions related to age, gender, monthly income, and education, of participants. The second section of the questionnaire was the Sindhi version of the GMAS that was translated from the Urdu version of GMAS used previously in Pakistani patients (Naqvi et al., 2018; Naqvi et al., 2020a). The GMAS scale consisted of 11 items that ask about a person’s medicine-taking behavior. Each item has four options, and each option has a score, namely, always (0), mostly (1), sometimes (2) and, never (3). The maximum achievable score is 33. The cut-off for designating a patient as adherent is 27. It is based on the scoring criteria described elsewhere (Naqvi et al., 2020a). The GMAS and its versions are available from the developer, but with the requirement of obtaining permission.

### 2.4. Translation process

The GMAS scale was translated from Urdu to Sindhi by a native Sindhi speaker (T1) whose additional language was Urdu. The T1 was a healthcare professional with a postgraduate qualification in research and was a subject matter expert. This version prepared by T1 was termed as the first version V1. In addition, another translation was conducted by another native language speaker (T2) who was a non-subject matter expert and was blinded. T2 had a postgraduation qualification in business administration. The document prepared by T2 was termed as second version V2. Both drafts were reconciled at this point and harmonized to form one final document termed as third version V3. The translators T1 and T2 jointly conducted the harmonization. Any ambiguity was checked and rectified at this point. The V3 was translated back into Urdu language. The back translation was carried out by another native language speaker (T3), who was a healthcare professional with a bachelor’s degree and was blinded to the study.

All experts (T1, T2, and T3), hailed from diverse regions within the Sindh province, bringing their own regional Sindhi language dialects and linguistic variations. This ensured capturing regional dialects and linguistic variations harmoniously during the translation of the scale. Furthermore, special emphasis was laid on key terminologies related to linguistics, medical and technical equivalence. The translation process was completed at this point. The final document was termed V4 and later GMAS-S. After the translation, the scale was piloted in 5 patients before the actual study. This piloting was done to check for any problems in understanding of the scale items among participants. No language and comprehension issues were reported. At this stage, the process was deemed complete.

### 2.5. Data collection process

The data was collected after obtaining ethical clearance and was done so using the survey questionnaire. The data was collected based on OPD activity and peak visiting hours, i.e., evening hours. The surveys were handed over to the administrative staff and nurses, who offered it to the people attending the clinics. The data was collected once from each participant and no follow-up was carried out. Participants who agreed to participate and provided their consent were asked to fill in their response in the questionnaire. Patients were requested to provide their filled questionnaire within the duration of their stay at the OPD as it was not possible to collect their response later. All hard copies were securely disposed of once the data were digitized and verified.

### 2.6. Study outcomes

The primary outcome of this study was the attainment of satisfactory values for most of the fit indices for establishing validity. In addition, the attainment of an acceptable value for reliability was considered another outcome. The secondary outcome was the documentation of adherence score of the patients.

### 2.7. Data management and statistical analyses

The data was analyzed using IBM SPSS version 23. The data distribution was checked. The discrete data were expressed using mean (x¯) and standard deviation (SD). Reliability was assessed using Cronbach’s alpha and was considered satisfactory if it was ≥0.7 (Costa and Sarmento, 2019). Besides, intraclass correlation coefficient (ICC) values were reported. Additionally, the item-to-correlation (ITC) were also reported to assess the relationship between an individual item and the whole construct. A positive relationship was hypothesized.

Validity of the GMAS-S was assessed through confirmatory factor analysis (CFA). CFA was conducted using IBM AMOS version 25 with the formation of a structural equation model (SEM) for a three-factor model as indicated in the Urdu version of the scale earlier (Naqvi et al., 2020a). Fit indices, namely, goodness of fit index (GFI), adjusted goodness of fit index (AGFI), Tucker Lewis index (TLI), comparative fit index (CFI), and root mean square error of approximation (RMSEA) were observed. The factor validity was deemed established if the majority of fit indices were in the acceptable range, i.e., GFI, AGFI, TLI, CFI ≥0.9, and RMSEA <0.08.

### 2.8. Ethical clearance and consent

This study was granted ethical clearance by the Institutional Review Board of Dow University of Health Sciences (Ref: IRB-2372/DUHS/Approval/2022/751). The data was collected after obtaining permission from the hospital. Participants were briefed about the study objectives and were invited. Those who agreed to participate were provided with informed consent attached on top of the questionnaire. The nature of consent was yes/no format, i.e., they could tick the appropriate box on the consent form if they agreed to participate. Participants who agreed to participate were asked to proceed and fill in their response. They were informed that no personal information will be recorded, their participation was voluntary, and their decision to participate will not affect their routine care at the clinics.

## 3. Results

The data from a total of 150 surveys were analyzed in this study. The average age of participants was between 43–44 years ( $\bar{x}=43.63$ , SD = 7.86). Most participants (N = 87, 58%) were above 40 years, and identified themselves as males (N = 86, 57.3%). More than half of the target sample had a monthly family income less than PKR 25,000 (N = 88, 58.7%). More than a third of participants were graduates (N = 54, 36%). The mean $\left(\bar{x}\right)$ adherence score was 17.23 ± 6.40. Most patients appeared non-adherent (N = 121, 80.7%) (Table 1).

**TABLE 1:** Background characteristics of participants (N = 150).

Characteristics	Frequency	Percentage
Age (in year)		
Age up to 40 years	63	42.0
Age above 40 years	87	58.0
Gender (Identification)		
Male	86	57.3
Female	64	42.7
Monthly Income (in PKR & USD)		
PKR <25,000 (USD <90.84)	88	58.7
PKR 25000–35,000 (USD 90.84–127.18)	19	12.7
Between 35,001–50,000 (USD 127.18–181.68)	26	17.3
More than 50,000 (USD more than 181.68)	17	11.3
Education level		
No formal education	36	24.0
Higher Secondary	50	33.3
Graduate	54	36.0
Masters	10	6.7
GMAS-S Score interpretation		
Adherent ≥27	29	19.3
Non-adherent ≤26	121	80.7

1 USD, equals PKR, 275.21 at the time of this writing.

The overall reliability of the Sindhi version of GMAS was roughly 0.7, i.e., Cronbach’s alpha (α) = 0.696 to all 11 items. The α value decreased if any of the 11 items were deleted from analysis. No increase in α value was reported in item deletion. It remained between 0.65–0.7. The intraclass correlation coefficient (ICC) was reported at 0.696 (95% CI: 0.618–0.763). The corrected item-to-total correlations ranged from 0.203–0.474 indicating a positive relationship with the overall scale construct. The details are presented in Table 2.

**TABLE 2:** Reliability and internal consistency.

GMAS items	Corrected ITC	α if item deleted
1	0.276	0.689
2	0.316	0.681
3	0.320	0.680
4	0.293	0.683
5	0.444	0.658
6	0.472	0.654
7	0.474	0.657
8	0.349	0.675
9	0.446	0.660
10	0.203	0.695
11	0.217	0.693

**FIGURE 1:**
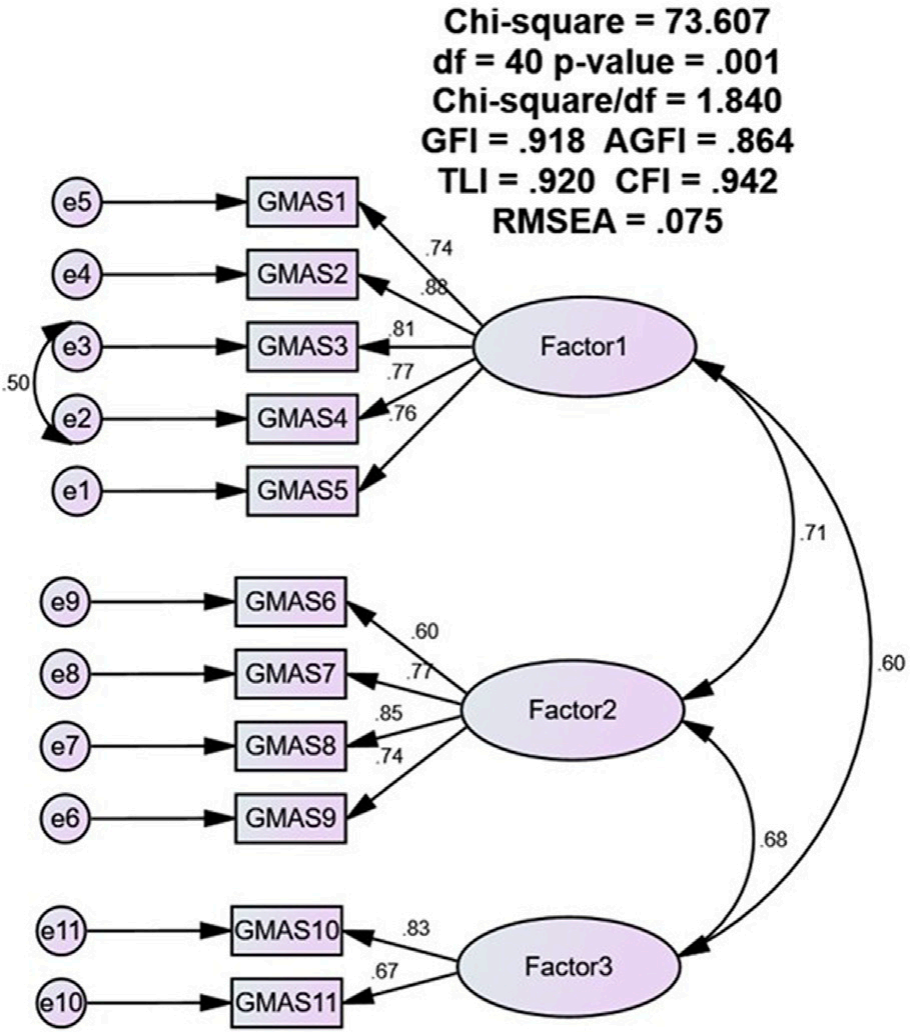
CFA path diagram.

The CFA analysis reported acceptable fit indices values for the 3-structure model. The χ2/df value was 1.84, and all other fit indices such as GFI, TLI, CFI were above 0.9 but lower than 0.95. The AGFI was lower than 0.9. The RMSEA value was 0.075, i.e., less than 0.08. The path diagram is presented as Figure 1.

## 4. Discussion

This study was aimed at translating and validating the Sindhi version of the scale in Sindhi speaking patients with chronic illnesses. Most participants were relatively young, identified themselves as male, and were educated. Many had a lower monthly family income. The Cronbach’s alpha value was approximately 0.696 that indicated an acceptable reliability for the Sindhi version of the scale. Available evidence reports that a value 0.7 for Cronbach’s alpha (α) is acceptable (Costa and Sarmento, 2019). In comparison, the reliability is less than the Urdu, Arabic, Nepalese and Vietnamese versions which reported an α value above 0.8 (Naqvi et al., 2018; Naqvi et al., 2020a; Naqvi et al., 2020b; Nguyen et al., 2021). Studies that have validated the English and Chinese versions of scale have reported α values of 0.74 and 0.781 respectively (Naqvi et al., 2019b; Wang et al., 2021). In addition, the α value decreased if any of the 11 items were deleted from analysis. This meant that all the items contributed positively to the consistency of the scale and assessed essential aspects of adherence. In some cases, the overall reliability of the scale may increase as a result of item deletion as reported in a study (Kim et al., 2016; Mahmoud et al., 2021). In previous two studies involving GMAS Urdu and Arabic versions, the items in the 3^rd^ construct of the scale have reported a minor increase (0.01) in α value during item deletion (Naqvi et al., 2018; Mahmoud et al., 2021). However, other studies involving the same versions did not report an increase in the same (Naqvi et al., 2020b; Shrestha et al., 2021).

Similar to the previous models our study evaluated the fitness of a 3-factor model and reported acceptable values for the fit indices. The 3-factor model was selected as it aligned with the theoretical consideration of the scale as well as the findings of previous studies. The χ^2^/df value was 1.84, and all other fit indices such as GFI, TLI, CFI were above 0.9 but lower than 0.95. The AGFI was lower than 0.9. The RMSEA value was 0.075, i.e., less than 0.08. The χ^2^/df value is the index that assesses the chi square value to the degrees of freedom. A lower value or a value closer to 1.0 indicates a better fit. The CFI and TLI are other indices which assess the fitness of the proposed model by comparing the data and the proposed model (Costa and Sarmento, 2019). The GFI is an index that measures the fitness of the observed data with proposed model. The AGFI is similar to the GFI, however it adjusts the GFI for the model (Baumgartner and Hombur, 1996).

Based on the available cut-off values, all the values except for the value of AGFI, were in acceptable range (Costa and Sarmento, 2019). However, it is also reported that the GFI and AGFI are affected by the sample size and may increase with an increasing sample size (Hooper et al., 2008; Mulaik et al., 1989; Kim et al., 2016). Therefore, it may be lower in our study as our sample size was small. It is mentioned in the literature that the GFI and AGFI shall not be banked upon solely for designating the fitness of a model (Hooper et al., 2008). However, this highlights that the model could be further improved with better sampling and a larger sample size.

Another finding was the stark imbalance between adherent and non-adherent patients. Previous studies, conducted in Pakistani population have highlighted percentage between 36% and 38% for non-adherence. However, >80% of patients appeared non-adherent in this study. Since there are no studies that have delved into adherence issues in this population, the results cannot be subjected to comparison. In addition, the observed percentage of non-adherent individuals cannot be conclusively attributed to random chance. However, there are implications as this finding highlights that most patients from this population do not follow the recommendations. This underscores the need to conduct large scale studies to evaluate the medication taking practices in this community and its determinants. This will inform future interventions to counter non-adherence in this population.

This study has few limitations. The sample size gathered for this study only satisfies the minimum requirements for statistical validation of a scale using the CFA technique. This may not be representative of the diversity and characteristics of the entire Sindhi-speaking patient population visiting the healthcare setting and therefore, should be interpreted with caution. Therefore, it is recommended to conduct future studies with larger sample sizes that would provide more robust and representative data. In addition, the study did not document the health profile of the patients in terms of specific diseases and medications. This would significantly limit the understanding of readers pertaining to the medication adherence behavior of this population. Hence, the demographic findings presented in this study may not fully capture the association of certain illnesses or medications with adherence behavior. Therefore, it is also recommended to document health profiles and medication information of patients from this population in future studies.

## 5. Conclusion

The results of the study demonstrate that the Sindhi version of the GMAS is a valid and reliable scale to measure adherence in Sindhi speaking persons with chronic diseases. This study would enable the clinicians to effectively monitor and evaluate medication adherence among patients with chronic illnesses from this linguistic community. Moreover, this study would also set a precedent for future studies aiming to adapt the GMAS into other regional languages of Pakistan.

## Data Availability

The original contributions presented in the study are included in the article/Supplementary Material, further inquiries can be directed to the corresponding author.
